# Indiana Veterinary College

**Published:** 1899-05

**Authors:** 


### COMMENCEMENT EXERCISES.

### INDIANA VETERINARY COLLEGE.

The commencement exercises were held at the college building, March 24, 1899. The principal address was delivered by J. W. Carson, M.D., and the diplomas presented by S. E. Crose, A.M., M.D., Dean of the Faculty, to the following: Joe M. Greiner, Frank W. Brewer, Oris A. Carson, Albert C. Deaver, Clarence Dunn, Archibald M. Scott, Charles E. Rice, W. J. Ridgway, W. W. Conner, and M. W. Scott.

After the exercises there was a banquet at the Walhall Hall, given by the Secretary of the College, Dr. L. A. Greiner, in honor of Joe M. Greiner, one of the graduates. The Alumni Association held its meeting after the exercises closed, and the graduates of 1899 were admitted to the Association.

